# Comment on ‘Association of Computed Tomography‐Derived Body Composition and Complications After Colorectal Cancer Surgery: A Systematic Review and Meta‐Analysis’ by Van Helsdingen et al.

**DOI:** 10.1002/jcsm.13679

**Published:** 2024-12-22

**Authors:** Rachana Mehta, Ashok Kumar Balaraman, Muhammed Shabil, Sanjit Sah

**Affiliations:** ^1^ Clinical Microbiology, RDC Manav Rachna International Institute of Research and Studies Faridabad India; ^2^ Dr Lal PathLabs ‐ Nepal Kathmandu Nepal; ^3^ Research and Enterprise University of Cyberjaya Cyberjaya Malaysia; ^4^ Center for Global Health Research, Saveetha Medical College and Hospital, Saveetha Institute of Medical and Technical Sciences Saveetha University Chennai India; ^5^ Department of Paediatrics, Dr. D. Y. Patil Medical College Hospital and Research Centre, Dr. D. Y. Patil Vidyapeeth Pune India; ^6^ Department of Public Health Dentistry Dr. D.Y. Patil Dental College and Hospital, Dr. D.Y. Patil Vidyapeeth Pune India

Dear Editor,

We read with great interest the article titled ‘Association of computed tomography‐derived body composition and complications after colorectal cancer surgery A systematic review and meta‐analysis’ and commend the authors for their rigorous and insightful systematic review and meta‐analysis on body composition measurements using computed tomography (CT) scans as predictors of complications following colorectal cancer surgery [1]. This research addresses a highly relevant clinical issue, providing valuable information to guide surgical decision‐making. While the article provides important findings, we believe there are some additional aspects that could further strengthen its impact and provide readers with an even more comprehensive understanding.

First, although the authors conducted a thorough risk of bias assessment using the QUIPS tool, the article does not mention whether a sensitivity analysis was performed based on study quality. We suggest conducting such an analysis to examine how excluding lower‐quality studies (e.g., those rated as having a high risk of bias) may impact the pooled results. Sensitivity analysis could help readers better appreciate the robustness of the findings and determine whether the conclusions are consistent across studies with varying levels of methodological rigour [2].

Second, the certainty of evidence presented in this study could have been evaluated using the GRADE (Grading of Recommendations, Assessment, Development, and Evaluation) framework. GRADE is widely recognized for systematically assessing the quality of evidence and the strength of recommendations in health care research. Including a GRADE assessment would allow the readers to understand the confidence in the results across different outcomes, especially given the variability in CT measurement methods and clinical endpoints considered in the studies [3]. This would also facilitate the translation of evidence into clinical practice by offering clarity on the reliability of the conclusions.

Third, while the authors rightfully address the risk of publication bias in the discussion, we recommend the inclusion of formal statistical methods to assess this risk. A funnel plot or DOI plot, alongside statistical tests such as Egger's regression or the trim‐and‐fill method, could provide more concrete evidence of the presence or absence of publication bias [4]. These methods would further substantiate the robustness of the meta‐analytic findings by ensuring that the results were not disproportionately influenced by small or positive‐result studies.

Furthermore, it might be valuable to explore subgroup analyses based on factors such as the specific CT measurement (e.g., visceral fat vs. sarcopenia), patient age, or cancer stage. These analyses could uncover potential variations in predictive utility across different patient populations, making the results more clinically actionable. We encourage the authors to consider the evolving landscape of artificial intelligence (AI) and machine learning in CT image analysis. Including a discussion on how future research could incorporate AI‐based methods for body composition analysis could enhance the clinical utility of these measurements by providing more precise and automated predictors of post‐surgical outcomes.

We congratulate the authors for their valuable contribution to the field. By incorporating sensitivity analysis based on study quality, GRADE assessments, publication bias evaluations, and further exploration of AI‐based predictive models, future work could offer even greater insights. We appreciate the opportunity to comment on this important study and look forward to future research in this area.

## Author Contributions

RM, AKB, and MS critically and provided comments on methodological aspects. SS and MS written the edited the draft.

## Ethics Statement

The authors have nothing to report.

## Consent

The authors have nothing to report.

## Conflicts of Interest

The authors declare no conflicts of interest.

## Data Availability

The authors have nothing to report.
